# Tapered Quantum Cascade Laser Achieving Low Divergence Angle and High Output Power

**DOI:** 10.3390/s25154572

**Published:** 2025-07-24

**Authors:** Zizhuo Liu, Hongxiao Li, Jiagang Chen, Anlan Chen, Shan Niu, Changlei Wu, Yongqiang Sun, Xingli Zhong, Hui Su, Hao Xu, Jinchuan Zhang, Jiang Wu, Fengqi Liu

**Affiliations:** 1 Institute of Fundamental and Frontier Sciences, University of Electronic Science and Technology of China, Chengdu 611731, China; zizhuo.liu@uestc.edu.cn; 2 State Key Laboratory of Electronic Thin Films and Integrated Devices, University of Electronic Science and Technology of China, Chengdu 611731, China; 3 Laboratory of Optoelectronic Materials Chemistry and Physics, Fujian Institute of Research on the Structure of Matter, Chinese Academy of Sciences, Fuzhou 350002, China; 4 Key Laboratory of Semiconductor Materials Science, Institute of Semiconductors, Chinese Academy of Sciences, Beijing 100083, China; 5 School of Physics, University of Electronic Science and Technology of China, Chengdu 611731, China; 6 Mozi Laboratory, Zhengzhou 450001, China

**Keywords:** quantum cascade laser, mid-infrared, optoelectronic device, semiconductor laser, tapered waveguide

## Abstract

In this work, we present a high-performance tapered quantum cascade laser (QCL) designed to achieve both high output power and low divergence angle. By integrating a tapered waveguide with a Fabry–Perot structure, significant improvements of tapered QCL devices in both output power and beam quality are demonstrated. The optimized 50 µm wide tapered QCL achieved a maximum output power of 2.76 W in pulsed operation with a slope efficiency of 3.52 W/A and a wall-plug efficiency (WPE) of 16.2%, while reducing the divergence angle to 13.01°. The device maintained a maximum power of 1.34 W with a WPE exceeding 8.2%, measured under room temperature and continuous wave (CW) operation. Compared to non-tapered Fabry–Perot QCLs, the tapered devices exhibited a nearly 10-fold increase in output power and over 200% improvement in WPE. This work provides a promising pathway for advancing mid-infrared laser technology, particularly for applications requiring high power, low divergence, and temperature stability.

## 1. Introduction

The mid-infrared (mid-IR) spectral region, ranging from 3 to 20 µm, is of significant importance due to its strong and unique molecular absorption features, generally referred to as “fingerprint” peaks, which enable highly sensitive and specific detection of gases and chemical compounds [1,2,3]. These features are crucial for applications such as environmental monitoring, industrial process control, medical diagnostics, and atmospheric sensing [4,5]. High-power, mid-IR lasers with low divergence angles are particularly desirable as they enhance light coupling efficiency and minimize energy loss over long distances. Consequently, the development of such laser sources has become a vital research area, forming the backbone of many mid-IR sensing and imaging systems [6,7]. Quantum cascade lasers (QCLs) have emerged as one of the most promising mid-IR laser sources, owing to their unique advantages. QCLs are semiconductor lasers that operate based on intersubband transitions in quantum wells, allowing for tailored emission wavelengths across the mid-IR and even terahertz (THz) regions through bandgap engineering [8,9,10,11,12]. QCLs have demonstrated several key benefits, such as high-power output, continuous-wave (CW) operation at room temperature, wide wavelength tunability, and compact device size [13,14,15]. These features make QCLs highly attractive for both fundamental research and practical applications in mid-IR spectroscopy and sensing.

Significant progress has been made in the development of QCLs over the past few decades, with the most common structure being the Fabry–Perot (FP) QCLs [16,17,18,19]. FP QCLs have demonstrated remarkable performance in terms of high-power laser output, achieving output powers of several watts and covering emission wavelengths from 3 to 12 µm [19,20]. The first room-temperature CW QCL with watt-level power output was developed by M. Razeghi et al. from Northwestern University in 2008, who reported a 4.6 µm QCL with an output power of 1.3 W and a wall-plug efficiency of 12.5% [21]. Building on this foundation, the same research team successfully demonstrated a QCL with a 400 µm ridge width that achieved a peak output power of 120 W at room temperature in 2009 [22]. More recently, in 2020, a CW output power of 5.6 W at room temperature was demonstrated for a QCL operating at 4.6 µm, representing the highest single-facet power achieved for a room-temperature CW QCL to date [23]. These achievements highlight the significant progress in enhancing the power and efficiency of FP QCLs.

Nevertheless, conventional FP QCLs still face limitations in mid-IR sensing applications, which hinder their further commercialization and broader adoption. One of the main challenges is the trade-off between output power and beam quality in FP QCLs. To achieve high output power, a wide ridge width is typically required, enabling high power but leading to multi-mode operation [24,25]. Conversely, a narrow ridge width is necessary to ensure the fundamental transverse mode operation, but this significantly reduces the output power and results in large beam divergence [26,27]. This inherent limitation of FP QCLs poses a significant challenge for applications that require both high power and low divergence, particularly in long-range gas sensing and high-resolution spectroscopy.

To address these challenges, tapered waveguides have been introduced as an effective solution. Tapered waveguides combine the benefits of a wide output facet and a broad gain section with a narrow ridge section, thus enabling high output power and low beam divergence simultaneously [28,29]. This design has been successfully implemented in various types of lasers, including conventional diode lasers and mid-IR QCLs, and has demonstrated significant improvements in both power and beam quality [30,31,32]. In 2007, researchers from the University of Würzburg demonstrated the feasibility of tapered QCLs, showing that a tapered structure could significantly reduce beam divergence while maintaining high output power [33]. A horizontal beam divergence of 6.6° with a peak output power of over 200 mW at room temperature for an emission wavelength of 8.9 µm was reported [33]. Then, in 2013, F. Capasso et al. reported a high-brightness tapered QCL with a peak output power of 3.8 W and a beam quality factor M^2^ of 2.25 at room temperature [32]. In the same year, this research group introduced a tapered QCL with a peak power of 4 W at room temperature, utilizing a combination of high- and low-reflectivity facet coatings to further enhance output power [34]. Subsequently, several research groups have conducted further studies to enhance the performance of tapered QCLs. For instance, in 2019, researchers from the Institute of Electron Technology in Warsaw reported a tapered QCL emitting at 4.5 µm with a convex geometry and a 1.7° taper angle, achieving a peak output power of 5 W and a horizontal beam divergence of 5.6° [35]. As the technology matured, researchers combined DFB QCLs with tapered waveguides, demonstrating a tapered distributed feedback (DFB) QCL with an output power of over 550 mW in CW operation at 293 K, while maintaining single-mode emission with a side-mode suppression ratio above 25 dB [36]. These studies collectively highlight the potential of tapered QCLs for high-power and low-divergence applications.

However, despite these impressive achievements, tapered QCLs still face several challenges. One of the main issues is that the increase in output power is not as significant as initially expected. Although several studies have reported peak output powers in the range of several watts, the increase in power is often limited by thermal effects and the complex structure of the QCLs [37,38]. Additionally, maintaining stable single-mode operation and low threshold currents remains a critical issue, especially as the taper width increases [39,40]. The thick upper waveguide layer in mid-IR QCLs requires precise etching techniques to achieve the desired taper profile without introducing significant losses or defects. In fact, reported tapered QCLs generally achieve a power increase of only around 2 to 5 times compared to their non-tapered counterparts. This suggests that the current design approaches are not fully leveraging the potential of tapered structures for power scaling. These challenges highlight the need for further research to optimize the design and fabrication processes of tapered QCLs.

In this work, we demonstrate an approach by integrating tapered waveguides with FP lasers on the same chip possessing a low divergence angle and high output power. By optimizing the waveguide width structure design, a significant enhancement in output power is achieved while effectively reducing the divergence angle. After completing the optimized device structure design and fabrication processes, the performance of tapered QCLs with different taper widths is systematically compared. It is identified that the 50 µm wide tapered QCLs exhibit optimal performance, with a maximum output power of 2.76 W in pulsed operation and a slope efficiency of 3.52 W/A, while achieving a maximum wall-plug efficiency (WPE) of 16.2%. The divergence angle is reduced to as low as 13.01°. Under room temperature and CW mode measurement, the tapered QCL achieves a maximum power of 1.34 W with a WPE exceeding 8.2%. The device also demonstrates excellent temperature stability, with characteristic temperature coefficients (T_0_ and T_1_) of 142.45 K and 294.98 K, respectively. Compared with non-tapered FP lasers, the tapered QCLs show a nearly 10-fold increase in power and over 200% improvement in WPE, while the substantial enhancement in output power is the highest value among the published results of tapered QCLs. This research highlights the significant potential of tapered waveguides in improving QCL performance and provides a foundation for the development of more efficient and versatile mid-infrared laser sources for various applications.

## 2. Materials and Methods

### 2.1. Material Epitaxy of QCL

The growth of high-quality QCL epitaxial materials is fundamental to this research, particularly the active region, which comprises multiple thin layers. The significance of the active region’s epitaxy lies in its ability to provide high-quality, strain-balanced layers, essential for achieving high-power, room-temperature CW operation.

**Figure 1:**
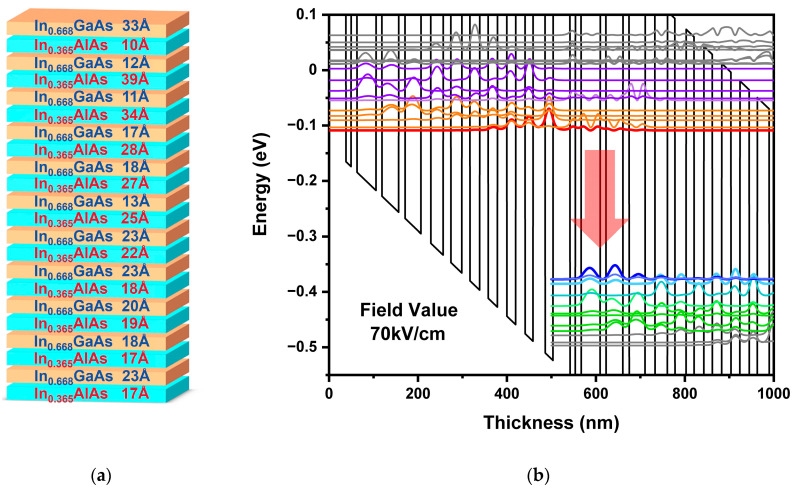
(**a**) Schematic of the epitaxial layer structure for one active region period in QCL; (**b**) conduction band diagram and wave functions of relevant energy levels of the active region under an electric field of 70 kV/cm.

The active region is designed based on the bound-to-continuum transition mechanism, which leverages the large density of states in the conduction band continuum to maximize optical transition efficiency. This design enhances the photon emission rate, thereby ensuring high-power output. Specifically, the design employs a double-phonon resonance approach, which is known for its high efficiency and robust performance in mid-infrared QCLs. The double-phonon resonance design allows for a more efficient energy transition between the upper and lower energy levels, thereby enhancing the overall efficiency of the laser. In this work, the active structure consists of 50 repetitions of periodic, multi-epitaxial layer groups, grown by molecular beam epitaxy (MBE) with strain-balanced In_0.668_GaAs/In_0.365_AlAs material compositions. As illustrated in Figure 1a, the layer sequence for one period in angstroms, starting from one In_0.668_GaAs quantum well, is as follows: 33/**10**/12/**39**/11/**34**/17/**28**/18/**27**/13/**25**/23/**22**/23/**18**/20/**19**/18/**17**/23/**17**. The In_0.668_GaAs quantum well layers are in regular font, the In_0.365_AlAs barrier layers are highlighted in bold, and two n-doped layers (1.5 × 10^17^/cm^3^) are underlined. This specific design ensures proper charge injection and population inversion while minimizing the net strain in the active region, thereby enhancing material quality and reducing dislocation density. Based on the specific epitaxial layer thicknesses of the active region shown in Figure 1a, as well as employing the single-band envelope function theory with effective mass approximation, the one-dimensional Schrödinger equation for electrons in the conduction band is solved. Utilizing the established MATLAB computational program (software version: MATLAB R2024b), the specific eigenvalues and eigenfunctions are calculated, corresponding to the subband bottom energies and respective wave functions for each well and barrier layer. Subsequently, the distribution of energy levels for the QCL active region is systematically organized and obtained. Figure 1b illustrates the calculated wave functions of the relevant energy levels in the conduction band reveal that the transition energy between the upper (Red) and lower (Blue) energy levels is 276 meV, corresponding to a lasing wavelength of 4.49 µm.

The QCL wafer utilized in this work is grown on an n-InP substrate (2 × 10^17^/cm^3^), with the active region serving as the core of the entire laser structure, sandwiched between two n-doped InP cladding layers. During the MBE growth of the active region, optimized growth parameters are used to ensure high-quality epitaxial layers with minimal defects. The growth conditions are carefully selected to balance the requirements of the different materials involved. Specifically, InAlAs tends to grow better at high substrate temperatures (495–530 °C) and low V/III ratios, whereas InGaAs grows better at low substrate temperatures (470–510 °C) and high V/III ratios. A compromise is reached by using the same substrate temperature and arsenic overpressure for both materials. Thus, the substrate temperature of 500 °C and the V/III ratio of 15–19 in growth are selected. The growth rates used are approximately 0.6 µm/h for In_0.668_GaAs and 1.05 µm/h for In_0.365_AlAs. The temperature stability of the group III effusion cells is calibrated to exceed 0.2 °C, and the group III flux stability is adjusted to exceed 1.2%. Given the sensitivity of the InGaAs layer to substrate temperature, the operation stability of the substrate heater at the growth temperature is confirmed to be better than 0.3 °C. These optimized growth conditions ensure uniform layer growth and high material quality, which are crucial for achieving high-performance QCL devices.

To further enhance the performance of the QCL, several optimization techniques are employed during the epitaxy process. First, the strain-balancing technique is used to minimize the net strain in the active region, thereby reducing dislocation density and improving material quality. Second, the growth parameters are fine-tuned to ensure optimal layer thickness and uniformity by adjusting the substrate temperature and V/III ratio to achieve the best possible growth conditions for both InAlAs and InGaAs layers. Additionally, the use of a high-thermal conductivity n-InP substrate is crucial for efficient heat dissipation and maintaining low operating temperatures.

### 2.2. Design of Tapered Structure

**Figure 2:**
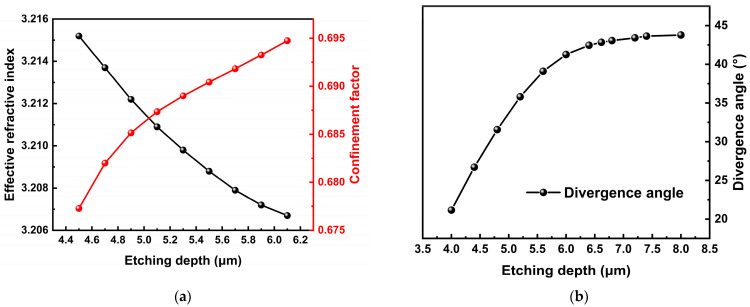
(**a**) The curves of simulated effective refractive index and confinement factor verse etching depth; (**b**) the calculation result of the relationship between etching depth and divergence angle.

To achieve effective operation in high-power fundamental transverse mode lasers, the laser structure must provide sufficient confinement to efficiently couple the laser while maintaining the fundamental mode output. As shown in Figure 2a, for a typical FP structure, the effective refractive index decreases while the confinement factor increases with increasing ridge waveguide etching depth. This indicates that deeply etched structures can offer better beam coupling and reduce losses in practical applications. However, deep etching also leads to an increase in divergence angle, as indicated in Figure 2b.

**Figure 3:**
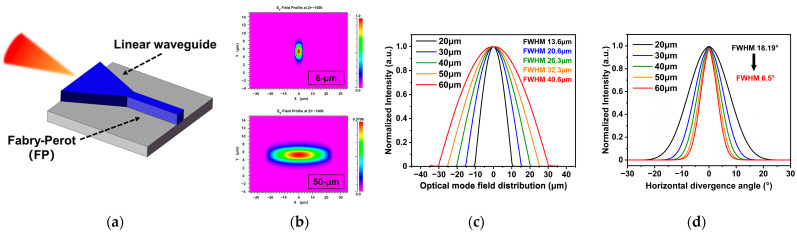
(**a**) Schematic diagram of the structure of each tapered QCL device; (**b**) simulated optical field distribution at the output facet of 6 µm and 50 µm wide tapered QCL devices; (**c**) the simulated results of the mode field size at the output end for different taper widths; (**d**) the relationship diagram between the slow axis divergence angle and the taper width at the output facet, with each device structure consisting of a 1 mm long FP section and a 1 mm long taper waveguide.

**Figure 4:**
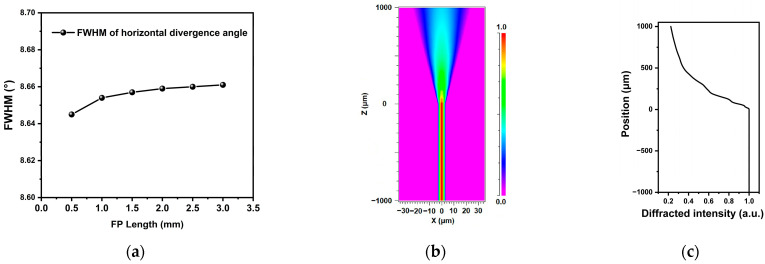
(**a**) The curve of the FWHM of the horizontal divergence angle for tapered QCLs with varying FP lengths; (**b**) the simulation result of optical field intensity distribution along the tapered QCL waveguide; (**c**) the distribution of fundamental mode diffraction intensity in the tapered QCL with 1 mm taper length and 50 µm taper width.

In this work, we adopt an integrated structure of FP and linear tapered waveguide to maintain high-power fundamental mode laser output while effectively controlling the divergence angle. As illustrated in Figure 3a, the tapered QCL consists of a narrow ridge FP section and a wider tapered waveguide section. The narrow ridge FP section provides a fundamental mode seed, which is then amplified and enhanced in the tapered waveguide without affecting the single-mode characteristic of the fundamental mode. In this work, the beam propagation method is employed to validate the fundamental mode characteristics through the calculation and simulation of the electromagnetic properties of various FP structures with different widths. For example, in a structure with a 6 µm wide FP section and a 50 µm wide tapered waveguide, the optical field distributions at the front and rear ends of the device (as shown in Figure 3b) remain consistent in terms of the fundamental mode distribution. The simulation results indicate that introducing a wider tapered waveguide does not disrupt the fundamental mode characteristic of the narrow ridge FP section but merely affects the optical mode size. Based on this model, the optical mode field distributions for different waveguide widths are summarized in Figure 3c. As the waveguide width increases, the near-field spot size also increases. By analyzing the near-field spot size, the corresponding divergence angles are calculated using Fourier transformation, as illustrated in Figure 3d. The horizontal divergence angle decreases with increasing output facet width, with the full-width at half-maximum (FWHM) horizontal divergence angle decreasing from 18.19° for a 20 µm wide facet to as low as 6.5°. Additionally, the length of the FP section may influence the overall device performance. However, as demonstrated in Figure 4a, the FP length has minimal impact on the beam quality of the tapered QCL device. In this work, both the tapered and FP sections are set to 1 mm in length.

The tapered QCL design enhances both beam quality and high-power output. The tapered structure increases the gain region area, allowing more carriers to participate in the stimulated emission process, while directly enhancing photon generation and achieving higher power output. Moreover, the linear tapered waveguide structure reduces the optical power density at the output facet. For example, with a 50 µm wide and a 1 mm long tapered section, as shown in Figure 4b,c, the fundamental mode diffraction intensity in the tapered amplification region decreases to 22%. This reduction mitigates spatial hole burning effects and delays the onset of self-focusing and filamentation, thereby facilitating higher output power.

### 2.3. Fabrication of Tapered QCL Devices

**Figure 5:**
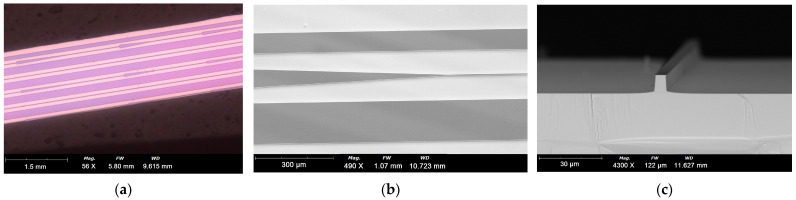
The scanning electron microscope (SEM) picture of (**a**) the tapered QCL samples with diverse waveguide structures arranged on the same wafer; (**b**) a single tapered QCL; (**c**) the cross-section of a FP section after dry etching.

In this work, samples with diverse waveguide structures were arranged on a single wafer (Figure 5a). Following the completion of the collective fabrication process, the wafer was cleaved into individual lasers and subsequently packaged for testing (Figure 5b). This approach ensures that samples with varying tapered structures share identical material and process conditions, thereby enhancing the reliability and comparability of the results. Specifically, all tapered QCL samples, regardless of their waveguide facet widths, possess the same ridge width in the FP section, which facilitates performance analysis and enables a focused investigation into the specific impact of structural design on laser performance.

The fabrication process of QCLs is detailed as follows: Initially, a 400 nm thick SiO_2_ layer was deposited on the sample surface via plasma-enhanced chemical vapor deposition. Subsequently, the SiO_2_ was patterned into the designed tapered QCL shape using photolithography and reactive ion etching (RIE) to form a hard mask. To achieve the desired etching depth for efficient coupling, deep etching was continuously optimized. The tapered waveguide and FP structures were fabricated using inductively coupled plasma (ICP) dry etching, followed by wet etching with an acid solution to attain an etching depth of approximately 8 µm. During the ICP dry etching process, the etching temperature was elevated to 260 °C to prevent the accumulation of etching products on the grating surface. Optimized etching parameters were employed, with the etching gas ratio of chlorine to argon set at 1:25, and the ICP RF and Table RF powers set at 300 W and 120 W, respectively. After dry etching, the sidewall profile was modified using a wet etching solution with a volume ratio of 16:30:6:300 for HBr:HCl:H_2_O_2_:H_2_O. The fabricated structure, as shown in Figure 5c, meets the design specifications, featuring smooth sidewalls that do not introduce significant losses. The SiO_2_ mask used for dry etching was then removed using a buffered oxide etch (BOE), and a new 200 nm thick SiO_2_ layer was deposited as an electrical isolation layer. Current injection windows were opened on the ridge top using photolithography and BOE etching. A top contact was formed, comprising a 30 nm Ti/20 nm Pt/300 nm Au layer deposited by e-beam evaporation and a 1 µm thick Au layer deposited by electroplating. After thinning the wafer to 100 µm and polishing the backside, an AuGeNi/Au layer was evaporated as the bottom contact and annealed at 370 °C for 1 min. The wafer was then cleaved into 2 mm bars (including 1 mm FP and 1 mm tapered waveguide). A high-reflectivity coating was applied to the back facet of the FP structure, and an anti-reflection (AR) coating was deposited on the output facet of the tapered waveguide. For device packaging, the chips were flip-chip soldered onto diamond submounts to enhance heat removal efficiency and subsequently mounted on copper heat sinks with indium solder, followed by wire bonding.

## 3. Results

**Figure 6:**
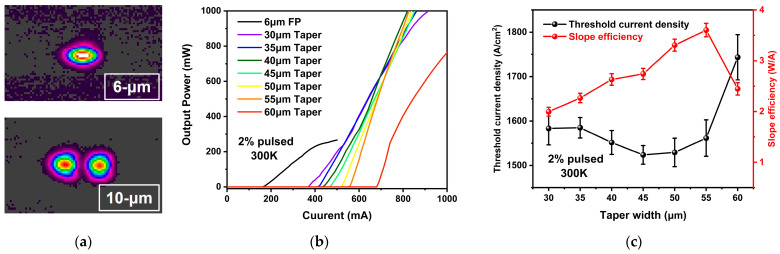
(**a**) The spot images of the 6 µm and 10 µm ridge width QCL devices; (**b**) the pulsed L-I characteristics of tapered QCL devices with different taper widths and a 6 µm wide FP QCL; (**c**) the curves of taper width versus threshold current density (Black) and slope efficiency (Red).

To ensure fundamental transverse mode operation of the FP section, two FP QCLs with ridge widths of 6 µm and 10 µm, respectively, and an etching depth of 8 µm were initially fabricated. The light beam spots measured using a beam quality analyzer are illustrated in Figure 6a for the 6 µm and 10 µm ridge width QCLs, respectively. The spot images confirm that the 6 µm ridge width FP QCL operates in the fundamental transverse mode, whereas the laser transitions to multi-mode operation when the ridge width is increased to 10 µm. Consequently, the FP section of the subsequent tapered QCL samples was designed with a 6 µm ridge width to serve as a fundamental mode seed source.

Based on the 6 µm ridge width FP section, tapered QCL samples with different waveguide widths were fabricated. Output power testing was conducted by securing the laser on a water-cooling platform with a chip facet positioned close to a calibrated thermopile detector. The peak power in pulsed operation (pulse width 1000 ns, repetition rate 20 kHz) was determined by dividing the average power by the duty cycle (2%). The temperature was monitored using a thermistor and regulated by a thermoelectric cooler (TEC). Figure 6b displays the pulsed power-current curves of tapered QCL devices with different waveguide facet widths (ranging from 30 µm to 60 µm in 5 µm increments) and a 2 mm cavity length. The heat sink temperature was maintained at 300 K (room temperature). The 6 µm ridge width FP QCL lases at an injection current of 180 mA and achieves a maximum power of 275 mW at 500 mA. The tapered QCL samples, which feature a larger active area than the FP lasers, exhibit higher slope efficiency and higher laser output power but have a higher threshold current than the FP QCL, as shown in Figure 6b. Moreover, as the waveguide width increases, the device threshold current continues to rise, and the slope efficiency also increases. The slope efficiency and threshold current density of each device were calculated from the power-current test results and are presented in Figure 6c. The slope efficiency of the tapered QCL increases with increasing taper width, reaching a peak value of 3.52 W/A at a ridge width of 55 µm, and then declines significantly. In contrast, the threshold current density decreases gradually with increasing taper width, from 1580 A/cm^2^ at a 30 µm taper width to 1547 A/cm^2^ at a 50 µm taper width. However, when the taper width exceeds 50 µm, the threshold current density increases sharply, exceeding 1750 A/cm^2^ at 60 µm. The decrease in slope efficiency and the increase in threshold current density in wider taper width devices may be attributed to thermal accumulation in the center of the device, which degrades the overall performance of the laser. This finding underscores the importance of investigating thermal distribution and thermal stability in tapered QCLs. Owing to the uniform epitaxial materials and device fabrication processes employed, devices with identical structures exhibit highly consistent performance, demonstrating excellent uniformity and reproducibility. Consequently, the results presented in this work are representative of the most typical outcomes for each structure. Detailed statistical analyses of all independent devices with different structures, further validating the reproducibility of these results, are also provided in Figure 6c in the form of error bars.

**Figure 7:**
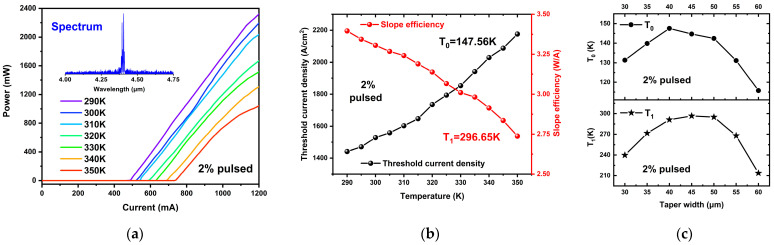
(**a**) The pulsed L-I characteristics of the 50 µm wide tapered QCL device at different temperatures, and the spectrum result of this device measured under 300 K is inset. (**b**) The calculated results of temperature-dependent threshold current density (Black) and slope efficiency (Red) of the 50 µm wide tapered QCL device under 2% pulsed operation. (**c**) The curves of characteristic temperature coefficients of tapered QCL samples with various taper widths.

The temperature stability of the devices was assessed by examining the pulsed power-current characteristics of the tapered QCLs at various temperatures controlled by the TEC. The lasing spectrum of the 50 µm taper width tapered QCL at 300 K was captured using a Fourier transform spectrometer and is depicted as an inset in Figure 7a. The emission wavelength of the tapered QCL in this work, 4.4 µm, closely aligns with the designed wavelength of 4.49 µm, indicating precise control of the epitaxial layer thickness during the material growth process. As illustrated in Figure 7a, the temperature dependence of the 50 µm taper width tapered QCL was measured across a temperature range of 290–350 K (in 10 K increments). The threshold current of the 50 µm taper width tapered QCL increases from 490 mA at 290 K to 740 mA at 350 K. Using these temperature-dependent results, the slope efficiency and threshold current density of the device at different temperatures were computed and graphed in Figure 7b. As the temperature increases from 290 K to 350 K, the threshold current density of the 50 µm taper width tapered QCL increases from 1441 A/cm^2^ at 290 K to 2176 A/cm^2^ at 350 K, while the slope efficiency decreases from 3.4 W/A at 290 K to 2.74 W/A at 350 K. Based on these findings, the characteristic temperature of the device was deduced using an exponential function, where T represents the heat sink temperature and T_0_ and T_1_ are the characteristic temperature coefficients. The fitting results yielded T_0_ and T_1_ values of 142.45 K and 294.98 K, respectively. The characteristic temperature coefficients of samples with different structures are displayed in Figure 7c. As the taper width increases from 30 µm to 40 µm, both T_0_ and T_1_ exhibit an upward trend. However, when the taper width surpasses 50 µm, both coefficients experience a significantly decrease. The 40 µm tapered QCL sample has the highest T_0_ value of 147.56 K, while the highest T_1_ value of 296.65 K is attained in the 45 µm tapered QCL sample. These results suggest that a taper width design within the range of 40–50 µm provides favorable temperature stability.

**Figure 8:**
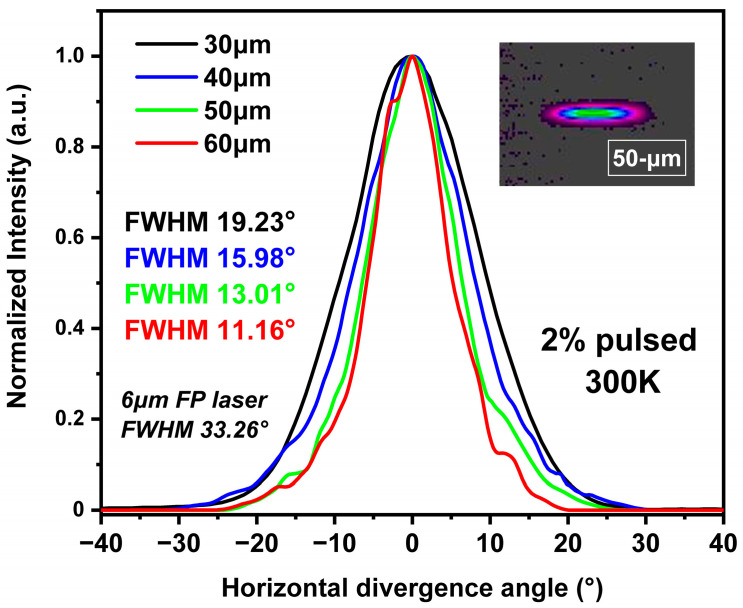
The horizontal divergence angle of different-width tapered QCLs measured by far-field profile along ridge width direction under room temperature and 2% pulsed operation. The attached figure shows the light beam spot of the 50 µm wide tapered QCL. The spot is measured by a pyroelectric camera placed 30 cm away from the laser after collimating.

Minimizing the divergence angle and enhancing beam quality are also crucial objectives in tapered QCL research. The beam quality of the QCL devices was assessed via far-field profile measurements taken along the ridge width direction. Figure 8 illustrates the divergence angles of samples with different taper widths measured by the far-field profile at room temperature (300 K) with a 2% pulsed injection current, which is 200 mA above the threshold current of each sample. The FWHM divergence angles for 30 µm, 40 µm, 50 µm, and 60 µm taper widths are 19.23°, 15.98°, 13.01°, and 11.16°, respectively. The divergence angle decreases with increasing taper width, in accordance with the theoretical simulation results presented earlier, although there is a slight deviation in the specific angles. Samples with taper widths up to 50 µm exhibited good single-mode characteristics, while the far-field profile of the 60 µm taper width sample tended towards a double-peak profile. This indicates that excessively wide taper widths fail to effectively filter out other transverse modes, thus not maintaining the fundamental mode characteristics as well as narrower tapers. The pyroelectric camera image of the light beam spot for the 50 µm taper width sample is shown in the top-right corner of Figure 8, revealing an elliptical intensity distribution that confirms stable single-mode lasing in pulsed operation.

**Figure 9:**
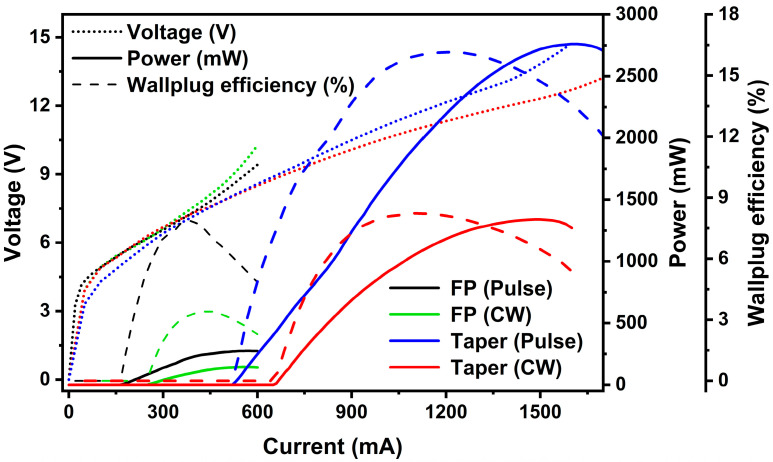
L-I-V characteristics of the 50 µm wide tapered QCL and 6 µm wide FP QCL measured at room temperature under both 2% pulsed and CW operation. The calculated WPE results are also included.

Considering output power, slope efficiency, characteristic temperature, and beam quality, the 50 µm wide tapered QCL device achieves a balanced performance in terms of high-power output, low divergence angle, and temperature stability, making it an optimal choice given the current QCL epitaxial material and device fabrication process. To quantify the improvements of the linear tapered QCL over the FP laser in terms of power, efficiency, and beam quality, LIV measurements were conducted in both pulsed and CW modes at 300 K for this sample and for the FP laser. The results are shown in Figure 9. In pulsed operation, the FP QCL has a power of 275 mW with a maximum WPE of approximately 7.7%, while the tapered QCL achieves a maximum output power of 2.76 W, which is nearly 10 times higher, with a maximum WPE of 16.2% near an injection current of 1100 mA. Under CW mode at room temperature measurement, the tapered QCL demonstrated significant performance advantages, with a maximum power of 1.34 W and a WPE exceeding 8.2%. This is in contrast to the FP QCL, which had a maximum power of only 144 mW and a maximum WPE of 3.4%.

## 4. Discussion

The objective of this work is to address the challenge of achieving high output power and high beam quality simultaneously in conventional FP QCLs. This is achieved by integrating FP structures with tapered waveguides on a single chip, resulting in a tapered QCL that delivers watt-level output power while maintaining a low divergence angle and fundamental transverse mode operation.

By fabricating and comparing devices with varying taper widths, it becomes evident that although wider taper widths theoretically offer higher gain areas, greater theoretical output power, and lower divergence angles, the experimental results do not always align with these expectations. Specifically, when the taper width exceeds 60 µm, the threshold current density of the devices increases rapidly, whereas both the slope efficiency and output power decrease. These observations, along with the lower characteristic temperature coefficients, indicate that overly wide taper widths could lead to increased threshold currents. This is attributed to the large gain area and potential thermal accumulation in the center of the taper region, which ultimately degrades performance. Moreover, overly wide taper widths can result in a larger cone opening angle. When this angle approaches or exceeds the divergence angle of the FP section’s fundamental mode, multiple modes are gradually excited, degrading beam quality. In contrast, a 50 µm taper width emerges as a more rational design. It avoids the thermal accumulation issues seen in wider tapers and, compared to narrower ones (e.g., 20 or 30 µm), achieves higher power and efficiency due to the larger gain region in the tapered waveguide. Additionally, the larger contact area between the tapered waveguide and the heat sink reduces thermal resistance, further improving heat dissipation and enhancing peak power output. Experimentally, this design achieved a high-power mid-infrared laser output of over 2.7 W with a divergence angle of 13.01°.

In terms of specific performance enhancements, comparisons between the tapered QCL and FP lasers of identical length and fabrication process revealed significant improvements. Under pulsed operation, the non-tapered QCL achieved a maximum output power of 275 mW, whereas the tapered QCL reached a maximum output power of 2.76 W, representing a nearly 10-fold increase. This substantial enhancement in output power is the highest reported to date. Moreover, the maximum WPE of the FP QCL was approximately 7.7%, while the tapered QCL achieved a maximum WPE of 16.2%, indicating an efficiency improvement of over 200%. Under CW operation, the performance gap is equally substantial: the tapered QCL achieved a maximum power of 1.34 W with a WPE exceeding 8.2%, compared with the non-tapered QCL’s maximum power of only 144 mW and a maximum WPE of 3.4%. Thus, in both pulsed and CW current injections, the tapered QCL demonstrated nearly a 10-fold increase in power and over 200% improvement in WPE. These substantial increases in power and efficiency can be attributed to several factors, as detailed below. The laser’s output power is derived from the difference between gain and losses. In the fabrication process, dry etching typically results in rougher sidewalls compared to wet etching, leading to higher losses. Additionally, there are inherent carrier injection losses within the material. Against this backdrop of high losses, the increase in gain results in a power boost that exceeds the proportional increase in the active area, thereby enhancing overall performance. Furthermore, the tapered waveguide structure effectively reduces the optical power density in the tapered region, thereby lowering the fundamental mode diffraction intensity and mitigating spatial hole burning and filamentation effects. This not only reduces optical losses within the laser but also minimizes the impact of sidewall roughness, thereby effectively lowering sidewall optical losses. Consequently, the tapered waveguide enhances gain while reducing various types of losses and offering better heat dissipation, all of which contribute to the significant increase in output power.

In recent years, as the performance requirements for mid-infrared lasers have increased, the design and fabrication of QCL devices have become increasingly complex. In such high-loss environments, the tapered QCL design presented in this work emerges as a more suitable candidate for high-performance mid-infrared applications, particularly where high output power, low divergence angle, and temperature stability are essential. This design is well suited for future high-precision and complex laser fabrication processes, making it highly significant for advancing the field of mid-infrared laser technology.

## 5. Conclusions

In summary, this work demonstrates that the monolithic integration of tapered waveguides with FP cavities significantly enhances QCL performance by overcoming the trade-off between power and beam quality. The optimized 50 µm device achieves remarkable performance: a peak pulsed power of 2.76 W with a wall-plug efficiency of 16.2%, a CW output power of 1.34 W with a wall-plug efficiency of 8.2% at 300 K, and a divergence angle of 13.01°. These achievements represent a tenfold increase in power and over 200% improvement in wall-plug efficiency compared to conventional ridge lasers. This is attributed to three critical mechanisms: modal volume expansion for enhanced photon extraction, reduced optical density to suppress nonlinear losses, and superior thermal management, as evidenced by the characteristic temperatures T_0_ = 142 K and T_1_ = 295 K. This advancement establishes tapered QCLs as high-performance mid-infrared sources for applications requiring watt-level power with near-diffraction-limited beam quality.

## Data Availability

The data presented in this study are available on reasonable request from the corresponding author.
